# Trends in prevalence and incidence of chronic respiratory diseases from 1990 to 2017

**DOI:** 10.1186/s12931-020-1291-8

**Published:** 2020-02-11

**Authors:** Min Xie, Xiansheng Liu, Xiaopei Cao, Mingzhou Guo, Xiaochen Li

**Affiliations:** 1 Department of Pulmonary and Critical Care Medicine, Tongji Hospital, Tongji Medical College, Huazhong University of Science and Technology, 1095 Jiefang Avenue, Wuhan, 430030 China; 2 Key Laboratory of Respiratory Diseases, National Ministry of Health of the People’s Republic of China and National Clinical Research Center for Respiratory Disease, Wuhan, China

**Keywords:** Global, Chronic respiratory diseases, Chronic obstructive pulmonary disease, Asthma, Incidence

## Abstract

**Background:**

Chronic respiratory diseases (CRDs) are leading causes of morbidity worldwide. However, the spatial and temporal trends in prevalence and incidence of CRDs have not been estimated.

**Methods:**

Based on data from the Global Burden of Diseases, Injuries, and Risk Factors Study 2017, we analyzed the prevalence and incidence trends of CRDs from 1990 to 2017 according to age, sex, region and disease pattern. Furthermore, the correlations between the incidence and the World Bank income levels, sociodemographic index (SDI), and human development index (HDI) levels were analyzed to assess the factors affecting incidence.

**Result:**

The total number of CRD cases increased by 39.5% from 1990 to 2017, nevertheless, the age-standardized prevalence rate (ASPR) and age-standardized incidence rate (ASIR) showed decreasing trends. The ASIRs of CRD, chronic obstructive pulmonary disease (COPD), pneumoconiosis, and asthma decreased, whereas the ASIR of interstitial lung disease and pulmonary sarcoidosis increased during the past 27 years. Significant differences between males and females in the incidence rates of pneumoconiosis, interstitial lung disease and pulmonary sarcoidosis were observed. Elderly people especially suffered from CRDs, except for asthma. For COPD, the ASIR decreased from low-SDI regions to high-SDI regions. The ASIR of interstitial lung disease and pulmonary sarcoidosis in the high-SDI region was highest and have increased mostly. The ASIRs for pneumoconiosis and asthma were inversely related to the HDI.

**Conclusions:**

In 2017, CRDs were still the leading causes of morbidity worldwide. A large proportion of the disease burden was attributed to asthma and COPD. The incidence rates of all four types of CRDs varied greatly across the world. Statistically significant correlation was found between the ASIR and SDI/HDI.

## Background

As major public health issues worldwide, chronic respiratory diseases (CRDs), including chronic obstructive pulmonary disease (COPD), pneumoconiosis, asthma, interstitial lung disease and pulmonary sarcoidosis, impose appreciable socioeconomic burdens on individuals and societies. Compared with other noncommunicable diseases, such as cardiovascular disease, cancer, and diabetes, CRDs are seriously neglected [1].

The most important risk factors for CRDs have been identified and include tobacco use, exposure to indoor and outdoor pollutants, allergens, occupational exposure, unhealthy diet, obesity, physical inactivity and other factors [2]. Because of an accelerated aging population and increased exposure to risk factors, CRDs are becoming more prominent problems for all regions of the world. The epidemiology and disease burdens of CRDs vary substantially worldwide. Previous studies have estimated the prevalence of CRDs at the regional or national level but not at the global level [3–5]. Understanding the prevalence and incidence trends of CRDs is vital for improving the control and prevention of CRDs.

The Global Burden of Diseases, Injuries, and Risk Factors Study (GBD) 2017 provided a comprehensive assessment of burden of CRDs in 195 countries and territories [6]. Based on this important data source, we assessed the spatial and temporal trends in the prevalence and incidence of CRDs by age and sex from 1990 to 2017. It was estimated that the burden of chronic diseases, including CRDs, accounted for 80% of the total burden in developing countries [7]. A negative association of socioeconomic status with the prevalence in COPD was revealed [8]. Asthma prevalence was found to be highest in high-income regions [9]. Therefore, the correlations between the incidence and the World Bank income levels, sociodemographic index (SDI), and human development index (HDI) levels were analyzed to assess the factors that affect incidence, and that is an important extension of previous research.

## Methods

### Data source

The prevalence, incidence, age-standardized prevalence rate (ASPR) and age-standardized incidence rates (ASIR) of CRD including COPD, pneumoconiosis, asthma and interstitial lung disease and pulmonary sarcoidosis, in 195 countries and territories during 1990–2017 were obtained from the GBD 2017 dataset (available online). The World Bank classifies economies into four income groups: high, upper-middle, lower-middle and low. For the SDI, countries were classified as high-SDI regions, high-middle-SDI regions, middle-SDI regions, low-middle-SDI regions and low-SDI regions. The HDI data were collected from the Human Development Reports [10].

Cases of COPD, pneumoconiosis (including asbestosis, coal workers’ pneumoconiosis, silicosis and other pneumoconiosis), asthma, interstitial lung disease and pulmonary sarcoidosis were identified based on the International Classification of Diseases and Injuries-10 diagnostic codes and are listed in Additional file 1: Table S1. It should be noted that interstitial lung disease and pulmonary sarcoidosis category does not contain pneumoconiosis estimates in the GBD dataset.

### Statistical analyses

The standardized approach for estimating the sex- and age-specific prevalence and incidence from the GBD 2017 dataset has been previously described [6, 11]. Bayesian meta-regression with DisMod-MR 2.1 was used as the main method of estimation for each condition. We generated 95% uncertainty intervals (UIs) for all data reported.

The ASIR (cases per 100,000 population) and ASPR (cases per 100,000 population) from 1990 and 2017 were analyzed for each condition. The estimated annual percentage change (EAPC) of the ASIR was calculated using a generalized linear model with a Gaussian distribution [12].

The relationships between the ASIRs and the World Bank income levels, SDI, and HDI were assessed to explore the main factors influencing the incidence rates. The SDI and HDI were included as covariates for incidence, and the Spearman's rank-order correlation coefficients were used to measure the strength of the correlations between the ASIR and SDI, the ASIR and HDI, the EAPC of the ASIR and the change in the SDI between 1990 and 2017. The percentage change in SDI between 1990 and 2017 was calculated by the ratio of the SDI in 2017 to the SDI in 1990 for each country. A *P* value of less than 0.05 was regarded as statistically significant.

## Results

### Chronic respiratory diseases

**Fig. 1:**
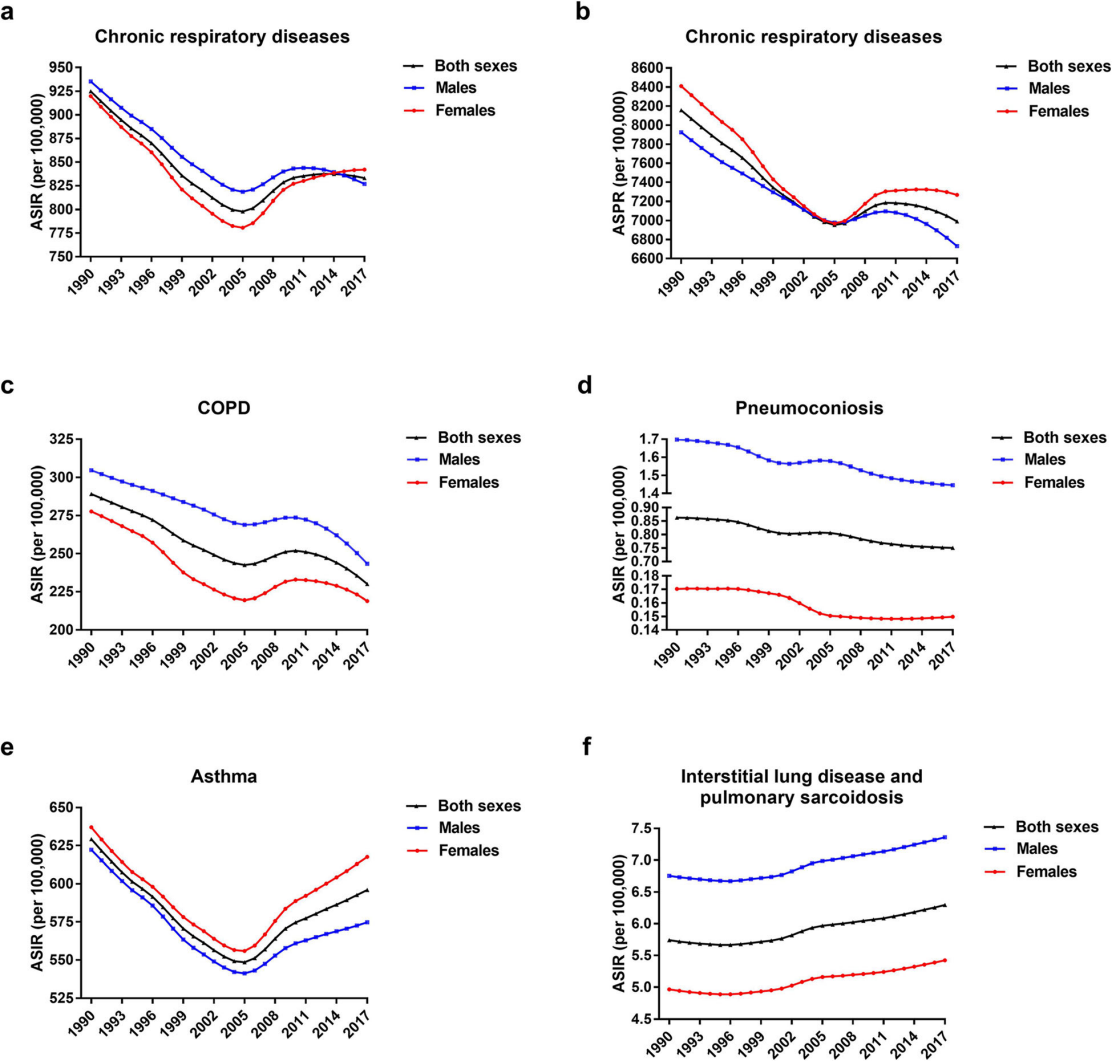
The age-standardized incidence rate (ASIR) and age-standardized prevalence rate (ASPR) of chronic respiratory diseases (CRDs) (**a**, **b**) including chronic obstructive pulmonary disease (COPD) (**c**), pneumoconiosis (**d**), asthma (**e**), and interstitial lung disease and pulmonary sarcoidosis (**f**) for both sexes (males and females). ASIR, age-standardized incidence rate; ASPR, age-standardized prevalence rate; COPD, chronic obstructive pulmonary disease

Globally, the total number of CRD cases increased by 39.5% from 389,713.8 (95% UI 362,943.0-416,351.4) thousand in 1990 to 544,899.2 (95% UI 506,937.5-584,858.4) thousand in 2017 (Additional file 2: Table S2). However, the ASIR decreased globally by an average of 0.33% (95% UI 0.17–0.48%) annually. Trends in the ASIR and ASPR of CRDs in both sexes are shown in Fig. 1. The ASIR decreased from 1990 to 2005 and subsequently increased from 2005 to 2017. The ASPR decreased from 1990 to 2005 and subsequently increased from 2005 to 2017 in females, whereas showed a decreasing trend in males from 2005 to 2017.

Interestingly, the ASIR of CRDs in males were higher than that in females until 2014, whereas the ASPR of CRDs in females were higher than that in males, which was attributed to the higher prevalence rates of COPD and asthma in females.

When analyzing each condition, an increase in the ASIR of interstitial lung disease and pulmonary sarcoidosis was observed, while the ASIRs of COPD, pneumoconiosis, and asthma decreased from 1990 to 2017. The lowest incidence rate of CRDs in 2005 was affected by the incidence trend of asthma.

### Chronic obstructive pulmonary disease

The number of COPD cases increased by 49.8% from 199,879.3 (95% UI 184,086.6-216,261.8) thousand in 1990 to 299,398.1 (95% UI 269,025.2- 330,073.8) thousand in 2017 (Additional file 2: Table S2). The ASPR in females was slightly higher than that in males in 1990 and 2017.

**Fig. 2:**
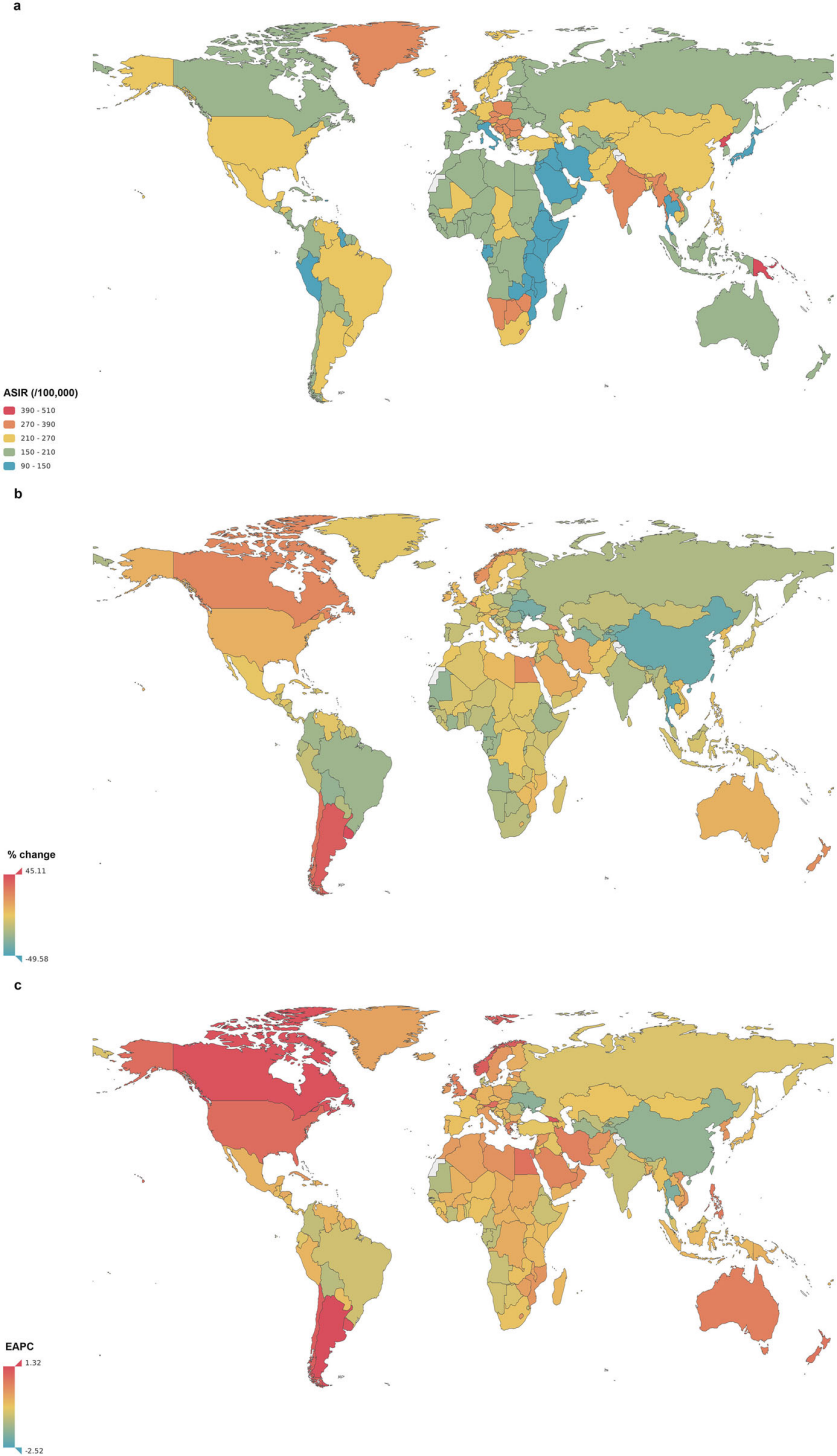
The global incidence of chronic obstructive pulmonary disease (COPD) for both sexes in 195 countries and territories. **a** The age-standardized incidence rate (ASIR) of COPD for both sexes combined in 2017. **b** The relative percentage change in the ASIR of COPD for both sexes between 1990 and 2017. **c** The estimated annual percentage change (EAPC) in the ASIR of COPD for both sexes from 1990 to 2017. COPD, chronic obstructive pulmonary disease; ASIR, age-standardized incidence rate; EAPC, estimated annual percentage change

Figure 2 shows the ASIR of COPD for both sexes combined in 2017. This rate varied greatly from 90.0 (95% UI 80.2–101.5) per 100,000 to 503.1 (95% UI 463.8–541.6) per 100,000 across the world. The countries with the three highest ASIRs were Papua New Guinea, North Korea and Lesotho. The lowest ASIRs were reported in Cape Verde, Singapore and Japan (Fig. 2a).

From 1990 to 2017, the ASIR decreased by an average of 0.68% (95% UI 0.55–0.81%) in both sexes globally. Significant decreases in the ASIRs were observed in the Maldives (EAPC = -2.52, 95% UI − 2.64 to − 2.40), Singapore (EAPC = -1.94, 95% UI − 2.15 to − 1.70) and Thailand (EAPC = -1.91, 95% UI − 2.02 to − 1.79) (Fig. 2c). The largest increases in the ASIRs were observed in Argentina (EAPC = 1.32, 95% UI 1.14–1.50), Uruguay (EAPC = 1.25, 95% UI 0.80–1.69) and Canada (EAPC = 1.24, 95% UI 0.74–1.75).

**Fig. 3:**
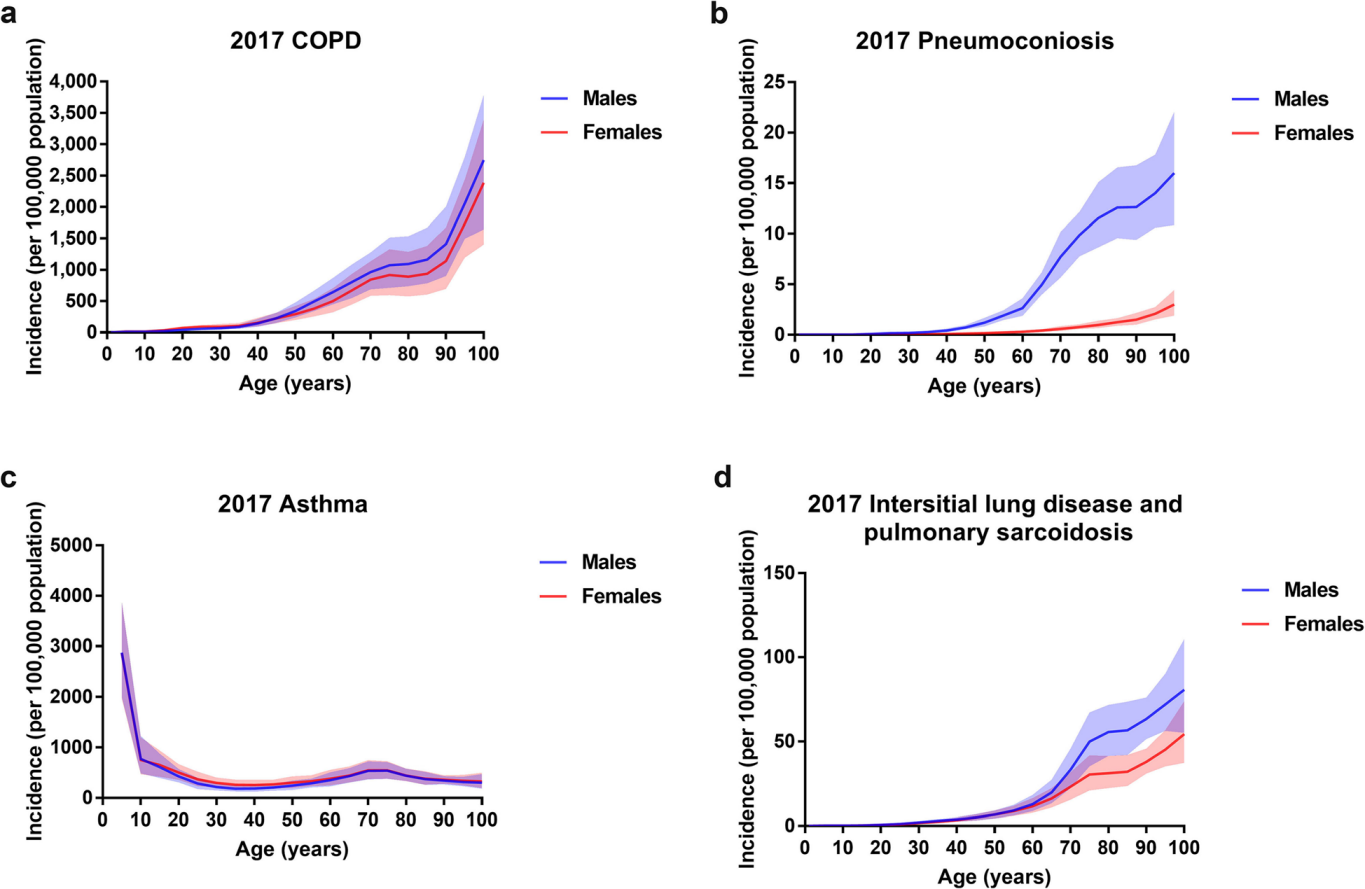
The global incidence rate of chronic obstructive pulmonary disease (COPD) (**a**), pneumoconiosis (**b**), asthma (**c**) and interstitial lung disease and pulmonary sarcoidosis (**d**) by age and sex in 2017. Shading shows 95% uncertainty intervals. COPD, chronic obstructive pulmonary disease

The global incidence rates of COPD in 1990 and 2017 by age and sex are shown in Additional file 3: Figure S1 and Fig. 3. COPD incidence increased with age, especially in those older than 90 years. The difference in the incidence rates between males and females was similar in 1990 and 2017, with a male predominance after age 40.

**Fig. 4:**
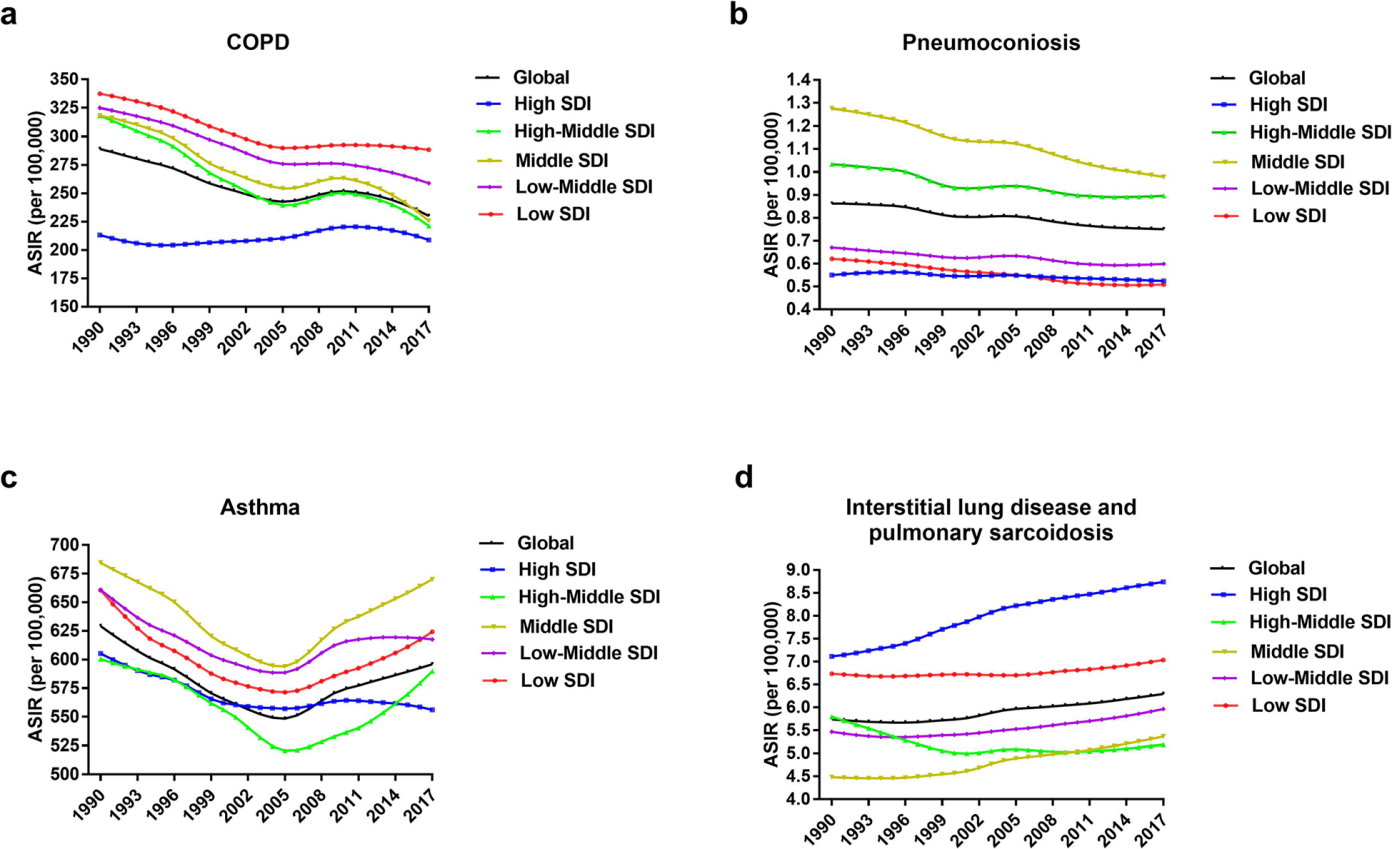
The age-standardized incidence rate (ASIR) of chronic obstructive pulmonary disease (COPD) (**a**), pneumoconiosis (**b**), asthma (**c**) and interstitial lung disease and pulmonary sarcoidosis (**d**) by sociodemographic index (SDI) regions from 1990 to 2017. COPD, chronic obstructive pulmonary disease; ASIR, age-standardized incidence rate; SDI, socio-demographic index

For SDI regions, the overall ASIR decreased from low-SDI regions to high-SDI regions. (Fig. 4). From 1990 to 2017, the ASIR had a slightly increasing trend in high-SDI regions (EAPC = 0.21, 95% UI 0.12–0.30) and declined gradually in the four other SDI regions. The largest decline was noted in high-middle-SDI regions (EAPC = -1.17, 95% UI − 1.36 to − 0.98). Similar decreasing ASIR trends were observed in low- and middle-income countries, and a slight increasing trend was observed in high-income countries according to World Bank income levels (Additional file 3: Figure S2). The ASIRs in high- and low-income countries were lower than those in middle-income countries which is not synchronized with the trends of ASIR grouped by SDI regions.

**Fig. 5:**
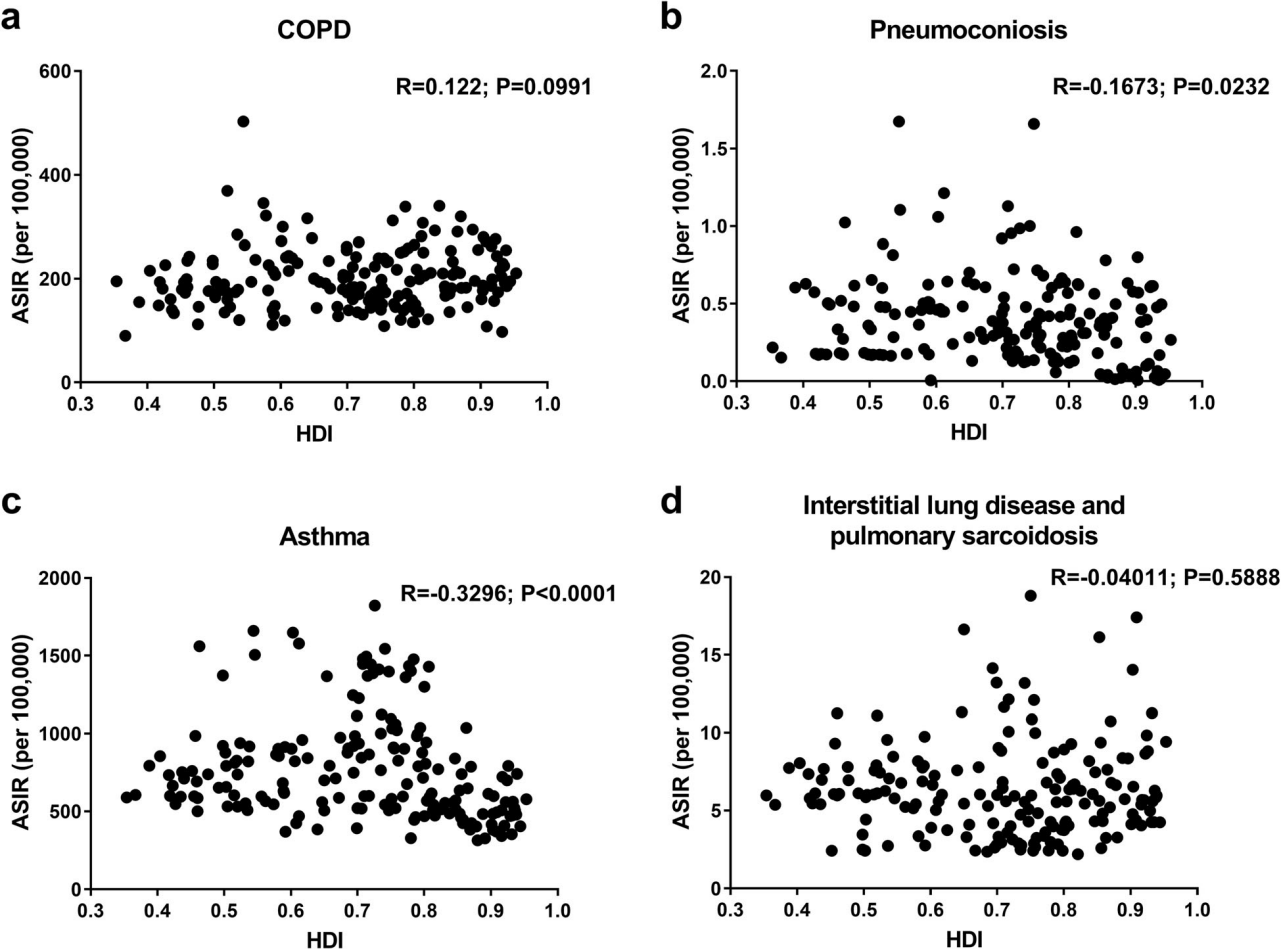
The correlation between the human development index (HDI) and age-standardized incidence rate (ASIR) of chronic obstructive pulmonary disease (COPD) (**a**), pneumoconiosis (**b**), asthma (**c**) and interstitial lung disease and pulmonary sarcoidosis (**d**) in 2017. HDI, human development index; ASIR, age-standardized incidence rate; COPD, chronic obstructive pulmonary disease

Neither the SDI nor HDI in 2017 were associated with the ASIR of COPD (Additional file 5: Figure S3; Fig. 5). Meanwhile, no correlations between the EAPC in ASIR and the change in SDI during 1990–2017 were observed (Additional file 6: Figure S4).

### Pneumoconiosis

**Fig. 6:**
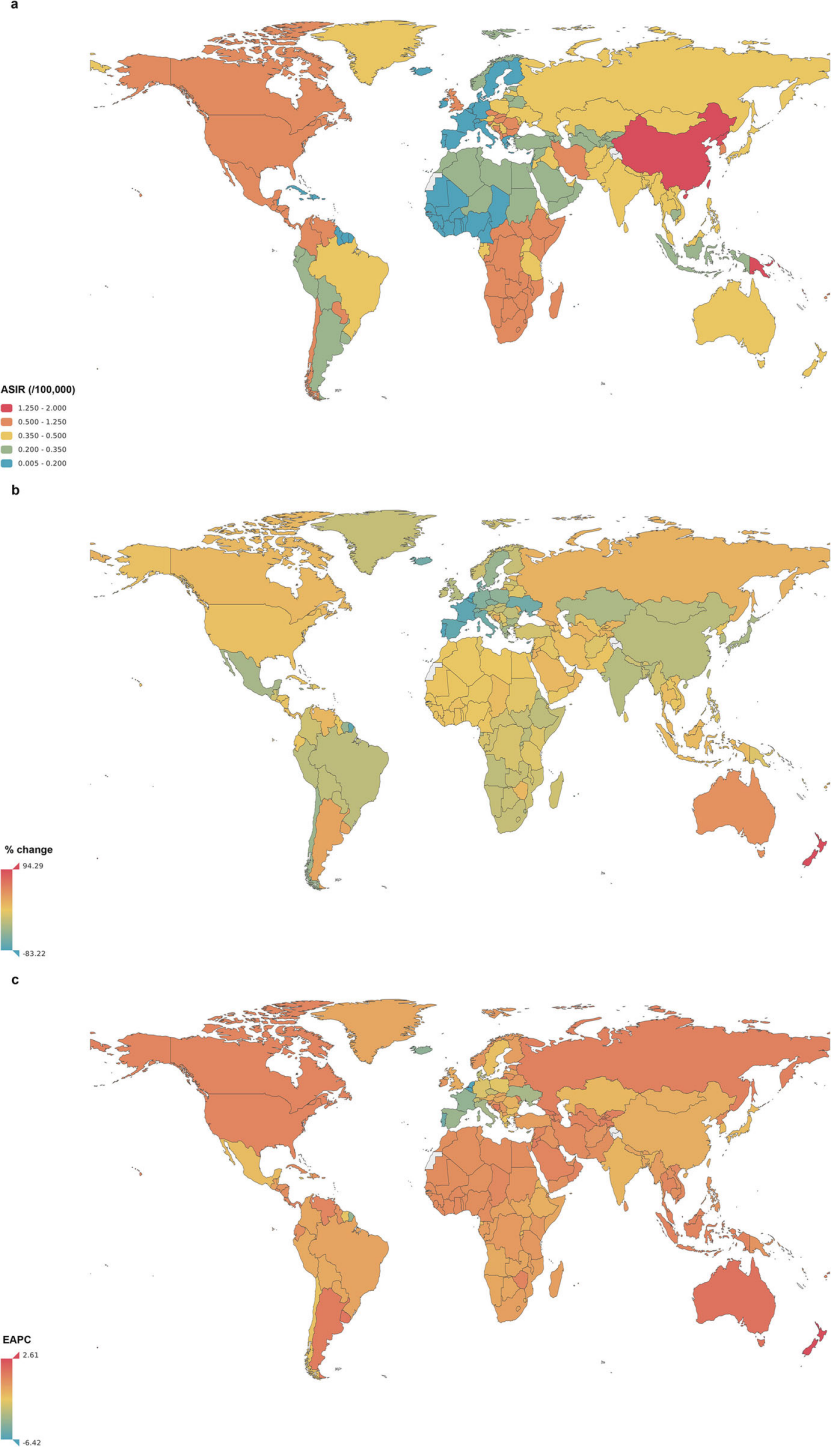
The global incidence of pneumoconiosis for both sexes in 195 conutries and territories. **a** The age-standardized incidence (ASIR) of pneumoconiosis for both sexes combinded in 2017. **b** The relative percentage change in the ASIR of pneumoconiosis for both sexes between 1990 and 2017. **c** The estimated annual percentage change (EAPC) in the ASIR of pneumoconiosis for both sexes from 1990 to 2017. ASIR, age-standardized incidence rate; EAPC, estimated annual percentage change

An increase of 81.1% in the number of pneumoconiosis cases were observed for both sexes from 1990 to 2017 (Additional file 2: Table S2), whereas the ASPR decreased slightly. The ASPR in males was significantly higher than that in females during the study period. Globally, the ASIR of pneumoconiosis ranged from 0.006 (95% UI 0.004–0.007) in Greece to 1.919 (95% UI 1.714–2.142) per 100,000 in Taiwan (China) in 2017 (Fig. 6a). New cases of pneumoconiosis increased by 66.0% from 36.2 (95% UI 32.5–40.0) thousand to 60.1 (95% UI 53.1–67.0) thousand from 1990 and 2017, with a downward trend in the ASIR (EAPC = -0.57, 95% UI − 0.53 to − 0.61). The largest ASIR increases were observed in New Zealand (EAPC = 2.61, 95% UI 2.55–2.67), Singapore (EAPC = 1.31, 95% UI 1.08–1.55) and Australia (EAPC = 1.25, 95% UI 1.16–1.33). Countries with the most pronounced decreases were the Netherlands (EAPC = -6.42, 95% UI − 7.01 to − 5.83), Belgium (EAPC = -6.30, 95% UI − 7.06 to − 5.55) and Portugal (EAPC = -5.51, 95% UI − 6.22 to − 4.79) (Fig. 6c). For each type of pneumoconiosis, the global ASIR for asbestosis showed an increasing trend (EAPC = 0.57, 95% UI 0.52–0.62) from 1990 to 2017, whereas the ASIRs for silicosis (EAPC = -0.77, 95% UI − 0.85 to − 0.70), coal workers’ pneumoconiosis (EAPC = -0.88, 95% UI − 0.93 to − 0.83), and other pneumoconiosis (EAPC = -0.50,95% UI − 0.54 to − 0.46) showed decreasing trends.

The incidence of pneumoconiosis increased with age in 1990 and 2017 (Additional file 3: Figure S1; Fig. 3). Significant sex difference was observed, and the incidence increased more among males than among females after age 20.

The ASIRs in high-SDI, low-middle-SDI, and low-SDI regions maintained low levels. The middle-SDI regions showed the highest ASIR and the greatest decrease over time (EAPC = -1.01, 95% UI − 1.05 to − 0.97), similar to the ASIR trend for upper-middle income countries (Additional file 4: Figure S2). A negative correlation between ASIR and HDI in 2017 was observed (R = -0.1673; *P* = 0.0232) (Fig. 5).

### Asthma

**Fig. 7:**
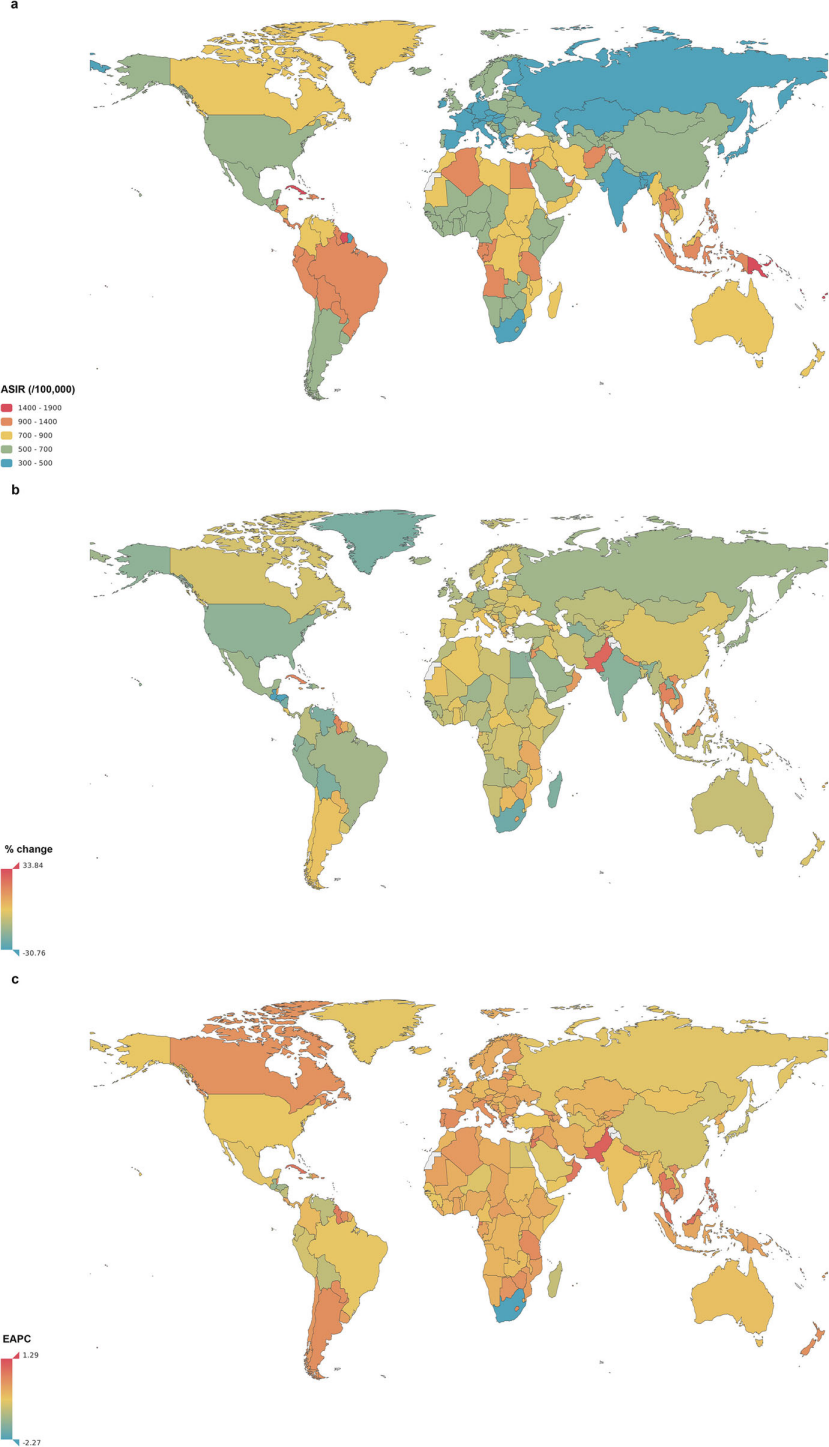
The global incidence of asthma for both sexes in 195 conutries and territories. **a** The age-standardized incidence rate (ASIR) of asthma for both sexes combinded in 2017. **b** The relative percentage change in the ASIR of asthma for both sexes between 1990 and 2017. **c** The estimated annual percentage change (EAPC) in the ASIR of asthma for both sexes from 1990 to 2017. ASIR, age-standardized incidence rate; EAPC, estimated annual percentage change

The number of asthma cases increased by 29.4% from 210,684.3 (95% UI 186,332.0-236,730.0) thousand in 1990 to 272,677.5 (95% UI 242,295.9-304,699.6) thousand in 2017 (Additional file 2: Table S2). In 2017, the ASIR of asthma differed considerably across the world, ranging from 314.1 (95% UI 268.2–363.0) per 100,000 to 1822.7 (95% UI 1484.7-2149.4) per 100,000. The three highest ASIRs were observed in Tonga, Puerto Rico and American Samoa, whereas the lowest ASIRs were observed in Italy, Spain, and Germany (Fig. 7a).

Asthma accounted for 69.4% of the total CRDs incidence cases in 2017. New cases of asthma increased by 19.0% from 1990 to 2017, although the ASIR remained relatively stable (EAPC = -0.18, 95% UI − 0.35 to − 0.01). The highest ASIR increases were observed in the Solomon Islands (EAPC = 1.29, 95% UI 1.14–1.44), Pakistan (EAPC =1.00, 95% UI 0.86–1.14) and Samoa (EAPC = 0.98, 95% UI 0.89–1.07). The greatest ASIR decreases were observed in South Africa (EAPC = -2.27, 95% UI − 2.92 to − 1.62), Guatemala (EAPC = -1.42, 95% UI − 1.57 to − 1.28) and Honduras (EAPC = -1.29, 95% UI − 1.42 to − 1.16) (Fig. 7c).

In 1990 and 2017, the global incidence rate of asthma decreased with age, especially in those younger than age 10. However, the incidence slightly increased in people aged 65–74 (Additional file 3: Figure S1; Fig. 3). The incidence rates in males and females were similar.

The temporal trends of the ASIRs in all five SDI regions exhibited decreases until 2005, followed by subsequent increases, creating a u-shaped pattern. The ASIRs in high-SDI and high-middle-SDI regions maintained low levels (Fig. 4), whereas the ASIR in low-income countries was extremely high (Additional file 4: Figure S2). The ASIR was found to be negatively related to the SDI (R = -0.3167; *P* < 0.0001) and HDI (R = -0.3296; *P* < 0.0001) in 2017 (Additional file 4: Figure S3; Fig. 5). Furthermore, there was an inverse correlation between the EAPC in the ASIR and the change of the SDI between 1990 and 2017 (R = -0.1552; *P* = 0.0303) (Additional file 6: Figure S4).

**Fig. 8:**
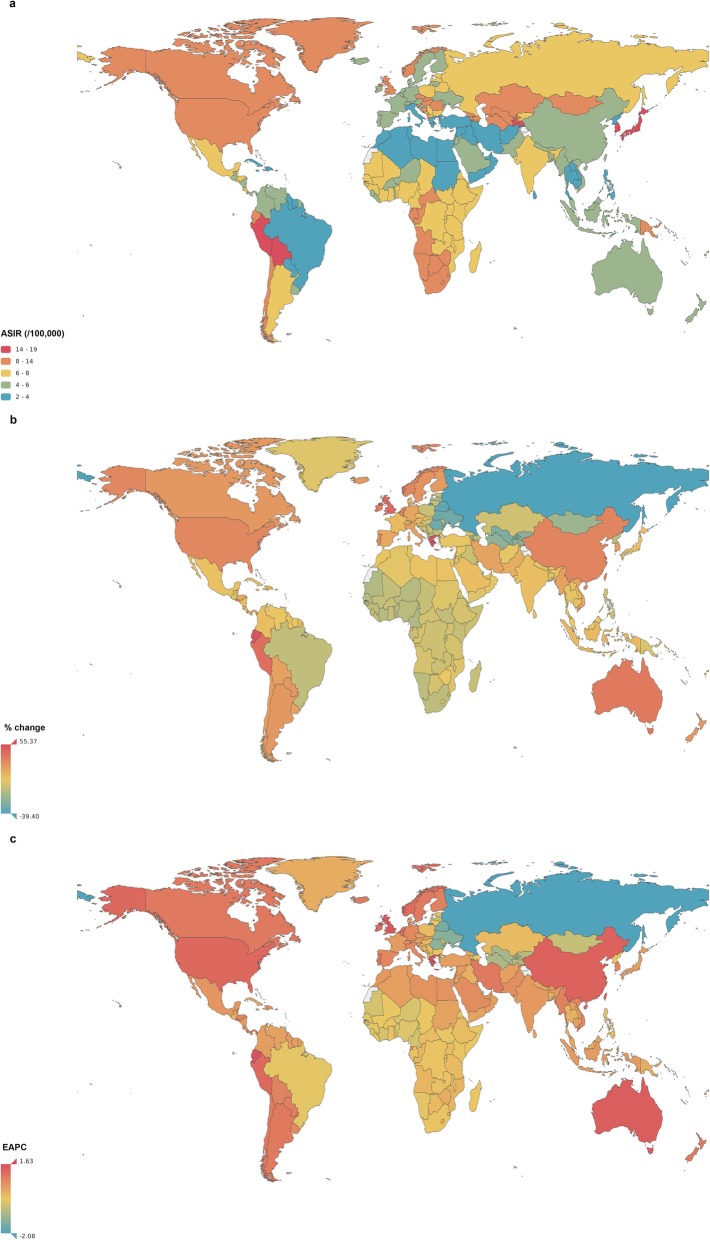
The global incidence of interstitial lung disease and pulmonary sarcoidosis for both sexes in 195 conutries and territories. **a** The age-standardized incidence rate (ASIR) of interstitial lung disease and pulmonary sarcoidosis for both sexes combinded in 2017. **b** The relative percentage change in the ASIR of interstitial lung disease and pulmonary sarcoidosis for both sexes between 1990 and 2017. **c** The estimated annual percentage change (EAPC) in the ASIR of interstitial lung disease and pulmonary sarcoidosis for both sexes from 1990 to 2017. ASIR, age-standardized incidence rate; EAPC, estimated annual percentage change

### Interstitial lung disease and pulmonary sarcoidosis

The number of interstitial lung disease and pulmonary sarcoidosis cases nearly doubled from 1990 to 2017 (Additional file 2: Table S2), whereas the ASPRs in both sexes slightly decreased.

The ASIRs of interstitial lung disease and pulmonary sarcoidosis varied more than nine-fold, ranging from 2.19 (95% UI 1.97–2.44) to 18.8 (95% UI 17.4–20.2) per 100,000, across the world in 2017. The highest rates were observed in Peru, Japan and Tajikistan and the lowest rates were observed in Oman, Iraq and Sudan (Fig. 8a).

Globally, the ASIR showed an increasing trend from 1990 to 2017, with an EAPC of 0.41 (95% UI 0.36–0.46) (Fig. 8c). The countries with the greatest increases were Greece (EAPC = 1.63, 95% UI 1.50–1.76), Ecuador (EAPC = 1.56, 95% UI 1.49–1.64) and the United Kingdom (EAPC = 1.48, 95% UI 1.45–1.52). The greatest ASIR decreases were observed in Russia (EAPC = -2.08, 95% UI − 2.25 to − 1.92), Belarus (EAPC = -1.60, 95% UI − 1.74 to − 1.46) and the Ukraine (EAPC = -1.42, 95% UI − 1.50 to − 1.33).

As shown in Additional file 3: Figure S1 and Additional file 5: Figure S3, the global incidence rates of interstitial lung disease and pulmonary sarcoidosis increased with age. The curves started to diverge after age 60, with the incidence increasing more in males than in females.

For the SDI regions, a downward ASIR trend was observed in only high-middle-SDI regions (EAPC = -0.35, 95% UI − 0.50 to − 0.20), and the most significant increase was noted in high-SDI regions (EAPC = 0.83, 95% UI 0.77–0.89). A similar trend of ASIR was observed in high-income countries (Additional file 4: Figure S2).

## Discussion

In 2017, CRDs were still the leading causes of morbidity across the world. A large proportion of the disease burden was attributed to asthma and COPD. From 1990 to 2017, the new and total cases of CRDs increased globally, whereas the ASIRs and ASPRs experienced decreasing trends, which may be explained by the population growth.

COPD was the most prevalent CRD worldwide in 2017, accounting for 54.9% of CRDs. The incidence rate of COPD increased with age in both sexes and decreased in all age groups from 1990 to 2017, except for elderly patients older than 80 years. At the national level, Japan ranked among the top three countries with the lowest ASIRs, whereas Papua New Guinea and North Korea had the highest ASIRs in 2017. Large inequities in COPD burden existed in different SDI regions. A significant decrease in the ASIR was observed in the high-middle-SDI regions. The bulk of the burden of COPD still occurred in the low-SDI regions.

Pneumoconiosis is mainly attributed to occupational risk factors, and a minimum proportion of cases may be caused by the natural environment or second-hand exposures [13]. Significant differences in incidence stratified by sex were observed for pneumoconiosis, which emerged as a male-predominant condition in people older than age 20. The sex-specific incidence of pneumoconiosis has a correlation with sex-specific occupational exposure. The temporal decline in the ASIR of pneumoconiosis was partly due to less occupational exposure. However, the ASIR for asbestosis showed an increasing trend, which might be partly explained by the long latency period between asbestos exposure and the manifestation of the disease [14]. Pneumoconiosis remains an important occupational health problem in developing countries. In 2017, the highest ASIRs were noted in China, Papua New Guinea and North Korea. The countries classified as middle-SDI and upper-middle income had the highest ASIRs and experienced significant decreases in the ASIRs from 1990 to 2017.

Asthma is a serious global health problem that affects all age groups, especially children, as shown in Fig. 3. From 1990 to 2017, the global ASIR showed a decreasing tendency that turned into a slight increase. In our study, the global ASIR of asthma decreased with age. According to the data from Human Development Reports, there was a significant decline in the global population aged younger than 5 from 1990 to 2000. The number of people aged older than 15 increased during 1990–2017. The young age (0–14) dependency ratio (per 100 people ages 15–64) showed a decreasing trend. On the other hand, the urban population of the world who have higher rates of asthma than people in rural areas, have grown rapidly from 1990 to 2017. Therefore, the U-shaped temporal ASIR trend for asthma might be a result of fertility decline, population aging and urbanization. There was an inverse correlation between the ASIR and the SDI/HDI in 2017. Furthermore, the EAPC in ASIR was negatively related to the change of the SDI between 1990 and 2017, indicating that the incidence of asthma could be partly attributed to social and economic factors.

Interstitial lung diseases are a group of diffuse parenchymal lung disorders [15] that are related to occupational or ambient air pollution exposures [16]. Pulmonary sarcoidosis is an inflammatory lung disorder associated with ethnicity and latitude [17]. Notably, there was an increasing trend in the global ASIR of interstitial lung diseases and pulmonary sarcoidosis from 1990 to 2017. The incidence in both sexes presented a steep rise after age 60 in both 1990 and 2017, and interstitial lung diseases and pulmonary sarcoidosis were much more prevalent in males than in females, suggesting that older males are particularly vulnerable. Although pulmonary sarcoidosis and lymphangioleiomyomatosis have a female predilection, [17] occupational exposure as a major cause of interstitial lung disease was more common in males than in females.

An age distribution analysis revealed that the incidence of CRDs, except for asthma, increased with age and exhibited a strong increase in elderly people aged over 90 years. According to the World Health Organization, 2 billion people worldwide are expected to be 60 years or older by 2050, accounting for 22% of the world’s population. Therefore, aging populations will become a profound challenge for CRD control and prevention.

Tobacco use is an important risk factor for CRDs. It was estimated that the prevalence of tobacco use would decline by 25% for males and 42% for females from 1980 to 2020, which may contribute to the decrease in the incidence of CRDs [18]. Sex and socioeconomic disparities in tobacco use have been identified. The cigarette smoking prevalence in males was more than four times higher than that in females worldwide [19]. Globally, most of the countries with the lowest prevalence of tobacco use were low-income and middle-income countries, and the highest prevalence in both males and females was concentrated in Europe and the western Pacific [20]. However, a sex or socioeconomic status disparity was not noted for the prevalence of CRDs or COPD; this result might be explained by other risk factors, including indoor air pollution, ambient air pollutant exposure, occupational particulate exposure.

A recent study revealed that the spatial heterogeneity of global particulate matter pollution significantly increased from 1998 to 2016. The most polluted areas were concentrated in developing middle-income regions, such as northern India, the central Indo-China Peninsula and southern and eastern China, which account for approximately 13.4% of the global land area and include 56% of the world’s population [21]. The CRDs cases exposed to risk factors such as tobacco use and air pollution were not available in the GBD 2017 dataset, which limited the assessment of the influence of environmental pollution on the prevalence of CRDs in this study.

The relationships between the incidences and the SDI/HDI were assessed. Notably, the prevalence of missed diagnoses of CRDs varied across all five SDI regions and countries with different HDIs, [22–25] thus affecting the estimated incidences of reported CRDs. In addition, the national epidemiologic survey on CRDs in many countries with large populations were not conducted in recent years, which may lead to an underestimation of the disease burden. Therefore, the results of this study could not accurately reflect the actual correlation between the incidences and the SDI/HDI in the real world.

## Conclusions

This study provided a global overview of CRDs in terms of prevalence and incidence and has important implications for further research and policy. From 1990 to 2017, the incidence rates of CRDs, COPD, pneumoconiosis, and asthma have decreased, whereas interstitial lung disease and pulmonary sarcoidosis showed an increasing trend. Elderly people especially suffered from CRDs, except for asthma. Significant sex differences were observed in the ASIR of pneumoconiosis, interstitial lung disease and pulmonary sarcoidosis. Large inequities in COPD burden existed in different SDI regions. The bulk of the burden of COPD still occurred in the low-SDI regions. The ASIR of interstitial lung disease and pulmonary sarcoidosis in the high-SDI region was highest and have increased mostly. The ASIRs for pneumoconiosis and asthma were inversely related to the HDI. The accelerated aging population and the increased exposure to risk factors including particulate matter pollution will increase the global burden of CRDs. The findings emphasized the need to take public health actions to reverse this impending disaster.

## Supplementary information

**Additional file 1:** **Table S1.** International Classification of Diseases and Injuries-10 (ICD-10) diagnosis code.

**Additional file 2:** **Table S2.** The global incidence, ASIR, prevalence and ASPR due to chronic respiratory diseases in 1990 and 2017.

**Additional file 3:** **Figure S1.** The global incidence rate of chronic obstructive pulmonary disease (COPD), pneumoconiosis, asthma and interstitial lung disease and pulmonary sarcoidosis by age and sex in 1990.

**Additional file 4:** **Figure S2.** The age-standardized incidence rate (ASIR) of chronic obstructive pulmonary disease (COPD), pneumoconiosis, asthma and interstitial lung disease and pulmonary sarcoidosis in countires classified by the World Bank income levels during 1990–2017.

**Additional file 5:** **Figure S3.** The correlation between socio-demographic index (SDI) and age-standardized incidence rate (ASIR) of chronic obstructive pulmonary disease (COPD), pneumoconiosis, asthma and interstitial lung disease and pulmonary sarcoidosis in 2017.

**Additional file 6:** **Figure S4.** The correlation between the change of socio-demographic index (SDI) and the estimated annual percentage change (EAPC) in the age-standardized incidence rate (ASIR) of chronic obstructive pulmonary disease (COPD), pneumoconiosis, asthma and interstitial lung disease and pulmonary sarcoidosis from 1990 to 2017.

## Data Availability

The datasets analyzed during the current study are available in the IHME Data (http://ghdx.healthdata.org/gbd-results-tool).

## Abbreviations

ASIR: Age-standardized incidence rate
ASPR: Age-standardized prevalence rate
COPD: Chronic obstructive pulmonary disease
CRDs: Chronic respiratory diseases
EAPC: Estimated annual percentage change
GBD: Global Burden of Diseases, Injuries, and Risk Factors Study
HDI: Human development index
SDI: Sociodemographic index
UIs: Uncertainty intervals
