# Supplementary material for: Trends in prevalence and incidence of chronic respiratory diseases from 1990 to 2017
Source: Respir Res. 2020 Feb 11;21:49. doi: 10.1186/s12931-020-1291-8 (PMC7014719; doi:10.1186/s12931-020-1291-8)
Supplement: Supplementary file 1 — Additional file 1: Table S1. International Classification of Diseases and Injuries-10 (ICD-10) diagnosis code. [file 12931_2020_1291_MOESM1_ESM.pdf]

Supplemental table 1. International Classification of Diseases and Injuries-10 (ICD-10) diagnosis code

Cause	ICD-10
Chronic obstructive pulmonary disease	J40-J44.9; J47-J47.9
Pneumoconiosis	J60-63.8; J65-J65.0; J92.0
Silicosis	J62-J62.9
Asbestosis	J61-J61.0; J92.0
Coal workers pneumoconiosis	J60-J60.0
Other pneumoconiosis	J63-63.8; J65-65.0
Asthma	J45-J46.9
Interstitial lung disease and pulmonary sarcoidosis	D86-86.2; D86.89-D86.9; J84-J84.9
