# Supplementary material for: Trends in prevalence and incidence of chronic respiratory diseases from 1990 to 2017
Source: Respir Res. 2020 Feb 11;21:49. doi: 10.1186/s12931-020-1291-8 (PMC7014719; doi:10.1186/s12931-020-1291-8)
Supplement: Supplementary file 2 — Additional file 2: Table S2. The global incidence, ASIR, prevalence and ASPR due to chronic respiratory diseases in 1990 and 2017. [file 12931_2020_1291_MOESM2_ESM.pdf]

Supplemental table 2. The global incidence, ASIR, prevalence and ASPR due to chronic respiratory diseases in 1990 and 2017

			**Chronic respiratory diseases**	**Chronic obstructive pulmonary disease**	**Pneumoconiosis**					**Asthma**	**Interstitial lung disease and pulmonary sarcoidosis**
					Overall	Silicosis	Asbestosis	CWP	Others		
**1990**	**No. incidence*10^3**		49341.4 (43429.9-55491.0) *	12824.8 (11943.8-13758.7)	36.2 (32.5-40.0)	15.0 (12.4-17.7)	4.3 (3.5-5.5)	9.8 (8.0-12.5)	7.0 (6.0-8.3)	36232.5 (30222.0-42178.5)	247.9 (226.6-270.1)
	**ASIR (per 100,000)**	**both sexes**	924.87 (828.62-1023.47)	289.10 (269.84-309.46)	0.86 (0.78-0.95)	0.36 (0.30-0.42)	0.10 (0.08-0.13)	0.24 (0.19-0.30)	0.17 (0.14-0.20)	629.17 (530.99-723.36)	5.74 (5.27-6.27)
		**male**	935.45 (840.71-1031.5)	304.76 (286.63-323.96)	1.70 (1.52-1.88)	0.74 (0.62-0.88)	0.17 (0.14-0.21)	0.50 (0.41-0.63)	0.29 (0.24-0.34)	622.24 (524.78-716.37)	6.76 (6,21-7.36)
		**female**	919.86 (821.49-1020.47)	277.64 (257.08-298.78)	0.17 (0.15-0.19)	0.04 (0.03-0.05)	0.05 (0.04-0.06)	0.02 (0.02-0.03)	0.06 (0.05-0.08)	637.08 (536.87-733.19)	4.97 (4.54-5.41)
	**No. prevalence*10^3**		389713.75(362943.03-416351.40)	199879.27(184086.58-216261.82)	291.19(257.76-328.29)	85.36(66.55-105.58)	47.13(35.78-63.43)	85.29(67.32-109.15)	73.41(60.31-88.43)	210684.26(186331.99-236729.869144)	3330.47(3037.41-3661.55)

		**both sexes**	8157.75(7663.98-8662.19)	4669.16(4296.67-5050.60)	6.97(6.18-7.87)	2.01(1.57-2.48)	1.11(0.85-1.48)	2.09(1.65-2.68)	1.77(1.46-2.12)	3981.75(3548.73-4417.55)	79.88(72.89-87.84)
	**ASPR (per 100,000)**	**male**	7926.45(7440.72-8419.84)	4618.39(4256.57-4984.11)	13.10(11.61-14.83)	3.94(3.07-4.90)	1.72(1.35-2.20)	4.34(3.43-5.60)	3.11(2.57-3.74)	3798.44(3386.57-4234.09)	87.99(80.30-96.54)
		**female**	8411.22(7889.66-8935.21)	4745.34(4353.84-5141.70)	1.96(1.69-2.29)	0.40(0.29-0.55)	0.62(0.45-0.89)	0.28(0.20-0.38)	0.66(0.53-0.82)	4168.66(3723.08-4614.65)	73.71(67.16-80.97)
	**No. incidence*10^3**		62161.3 (55134.8-69320.7)	18475.7 (16736.0-20255.6)	60.1 (53.1-67.0)	23.7 (19.1-29.0)	9.4 (7.7-11.6)	15.1 (12.0-19.8)	11.9 (9.9-14.3)	43123.4 (36191.5-50226.4)	502.2 (458.8-550.0)
		**both sexes**	833.08 (730.32-937.91)	230.07 (209.25-251.56)	0.75 (0.66-0.84)	0.30 (0.24-0.36)	0.12 (0.10-0.15)	0.19 (0.15-0.25)	0.15 (0.12-0.18)	595.97 (495.28-700.24)	6.30 (5.75-6.89)
**2017**	**ASIR (per 100,000)**	**male**	826.92 (725.52-932.84)	243.42 (222.31-265.37)	1.45 (1.28-1.62)	0.60 (0.48-0.73)	0.20 (0.17-0.24)	0.39 (0.31-0.50)	0.25 (0.21-0.30)	574.69 (474.57-678.50)	7.36 (6.71-8.04)
		**female**	842.09 (738.53-946.10)	218.97 (197.87-239.73)	0.15 (0.13-0.17)	0.03 (0.02-0.04)	0.05 (0.03-0.06)	0.02 (0.01-0.02)	0.05 (0.04-0.07)	617.55 (514.14-723.19)	5.42 (4.95-5.94)
	**No. prevalence*10^3**		544899.16(506937.52-584858.36)	299398.15(269025.16-330073.84)	527.46(470.01-593.19)	162.37(127.47-202.62)	92.15(73.11-118.32)	147.88(117.46-190.14)	125.07(104.97-149.25)	272677.53(242295.87-304699.57)	6234.16(5661.00-6848.17)

**ASPR (per 100,000)**	**both sexes**	6991.55(6496.73-7516.36)	3740.74(3369.84-4120.82)	6.63(5.91-7.44)	2.04(1.59-2.54)	1.15(0.93-1.48)	1.87(1.48-2.40)	1.56(1.32-1.86)	3599.59(3175.10-4039.91)	78.22(70.95-85.96)
	**male**	6731.06(6248.26-7267.53)	3711.47(3334.68-4101.16)	12.57(11.14-14.13)	4.04(3.16-5.02)	1.89(1.54-2.36)	3.88(3.07-5.02)	2.75(2.31-3.29)	3361.74(2951.90-3778.37)	86.87(78.75-95.25)
	**female**	7266.92(6741.78-7824.87)	3791.44(3430.97-4165.95)	1.62(1.40-1.88)	0.33(0.24-0.43)	0.53(0.39-0.75)	0.21(0.16-0.27)	0.54(0.44-0.67)	3833.87(3394.76-4292.91)	71.25(64.67-78.40)

*Data in parentheses shown as 95% UI.

ASIR, age-standardized incidence rate; ASPR, age-standardized prevalence rate; CWP, coal workers pneumoconiosis.
