# Supplementary figures and images for: Trends in prevalence and incidence of chronic respiratory diseases from 1990 to 2017
Source: Respir Res. 2020 Feb 11;21:49. doi: 10.1186/s12931-020-1291-8 (PMC7014719; doi:10.1186/s12931-020-1291-8)

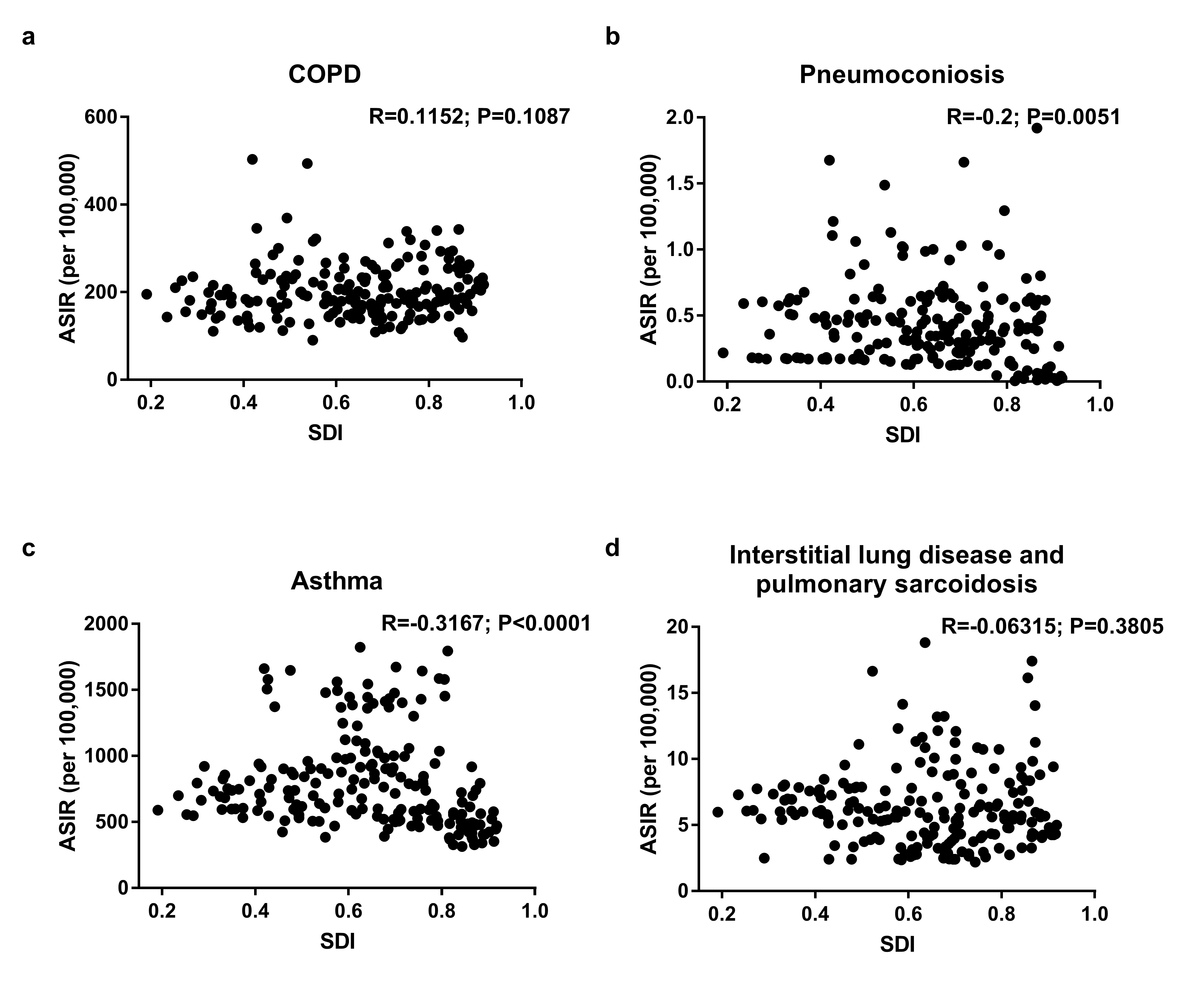

Supplement: Supplementary file 3 — Additional file 3: Figure S1. The global incidence rate of chronic obstructive pulmonary disease (COPD), pneumoconiosis, asthma and interstitial lung disease and pulmonary sarcoidosis by age and sex in 1990. [file 12931_2020_1291_MOESM3_ESM.tif]

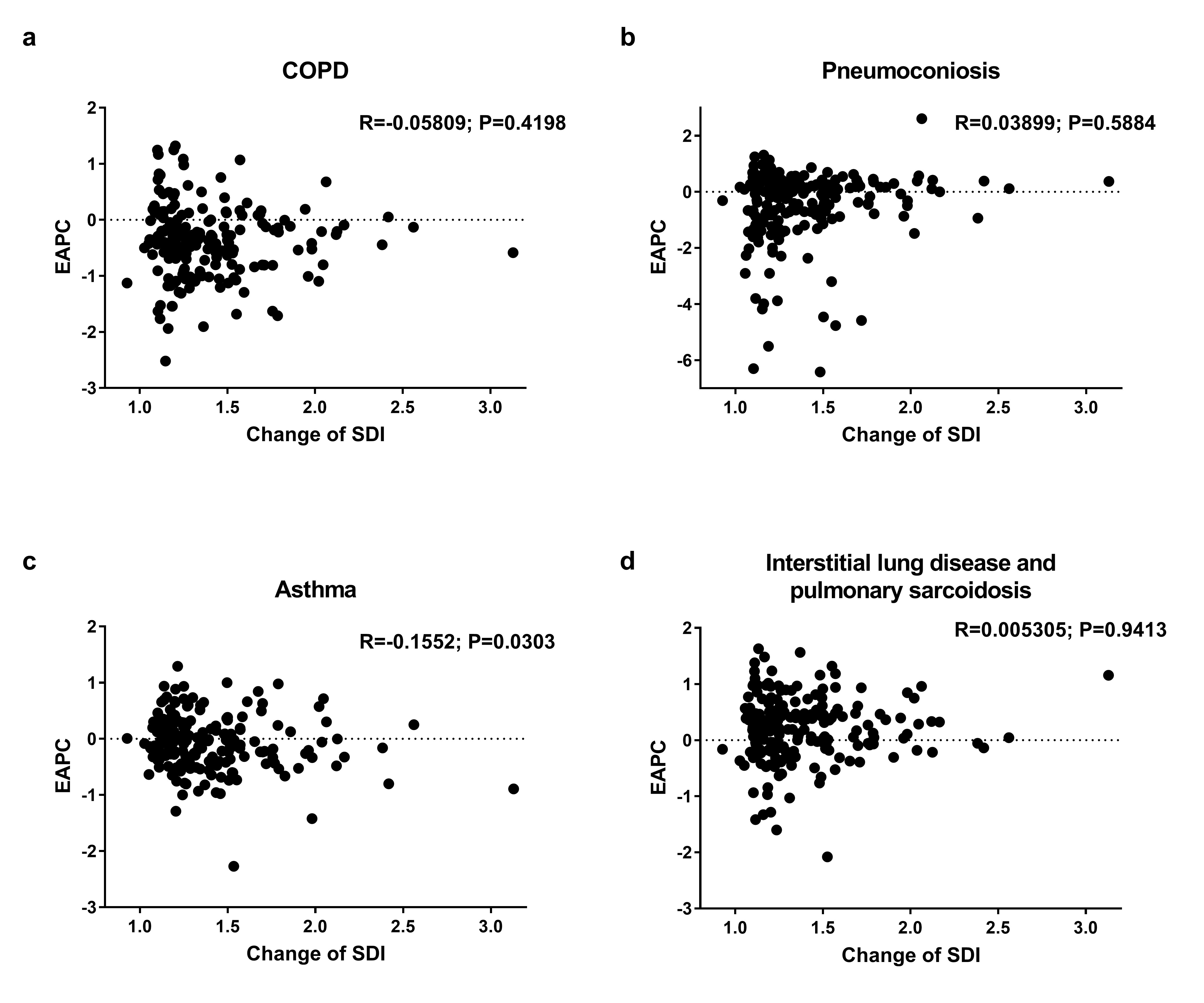

Supplement: Supplementary file 4 — Additional file 4: Figure S2. The age-standardized incidence rate (ASIR) of chronic obstructive pulmonary disease (COPD), pneumoconiosis, asthma and interstitial lung disease and pulmonary sarcoidosis in countires classified by the World Bank income levels during 1990–2017. [file 12931_2020_1291_MOESM4_ESM.tif]
